# Clinical Utility of the Adrenocorticotropin Stimulation Test with/without Dexamethasone Suppression for Definitive and Subtype Diagnosis of Primary Aldosteronism

**DOI:** 10.3390/ijms18050948

**Published:** 2017-04-30

**Authors:** Kosuke Inoue, Masao Omura, Chiho Sugisawa, Yuya Tsurutani, Jun Saito, Tetsuo Nishikawa

**Affiliations:** Endocrinology and Diabetes Center, Yokohama Rosai Hospital, Yokohama 222-0036, Japan; koinoue-tky@umin.ac.jp (K.I.); omura@yokohamah.johas.go.jp (M.O.); pochi_dog103@yahoo.co.jp (C.S.); yuya97tsuru1055@gmail.com (Y.T.); saitoj@yokohamah.johas.go.jp (J.S.)

**Keywords:** primary aldosteronism, adrenocorticotropin (ACTH) stimulation test, dexamethasone suppression test

## Abstract

The adrenocorticotropin (ACTH) stimulation test (AST) has been reported to be useful for diagnosing primary aldosteronism (PA), particularly for differentiating PA subtypes under 1-mg dexamethasone suppression (DS). The aim of our study was to clarify the effect of 1-mg DS on AST results. A retrospective cohort study was conducted using data for 48 patients (PA: 30/48). We estimated the difference in plasma aldosterone concentration (PAC) responsiveness to ACTH stimulation with single (AST alone) and combined (AST under 1-mg DS) tests within the same patient. We compared the diagnostic accuracy of these two tests for PA and the laterality of hyperaldosteronism. We found no differences in PAC responsiveness to ACTH stimulation between single and combined tests, and observed a significant positive linear relationship (30 min, *R*^2^ = 0.75, *p*-value < 0.01). Both tests showed the highest diagnostic accuracy for PA following 30 min of ACTH stimulation. The ability to detect the laterality of hyperaldosteronism was inconsistent and differed according to the two definitions: lateralization ratio and the absolute aldosterone levels in adrenal venous sampling. PAC responsiveness to ACTH stimulation was similar for AST with and without 1-mg DS. AST can be performed under both conditions with similar accuracy to detect PA.

## 1. Introduction

Primary aldosteronism (PA) is a major cause of secondary hypertension [1]. Detection of PA is critical because hyperaldosteronism increases the prevalence of cardiovascular complications and metabolic syndrome independent of blood pressure [2,3]. A plasma aldosterone concentration (PAC)/plasma renin activity (PRA) ratio (ARR) is generally used to screen hypertensive patients and confirmatory tests are subsequently performed to make PA diagnosis [1,4]. Adrenal venous sampling (AVS) is needed to clarify the laterality of hyperaldosteronism when patients meet the criteria of PA and they wish the surgical treatment if possible [1,4,5]. However, AVS has several disadvantages including invasiveness, high cost, and difficulty to perform the procedure.

Adrenocorticotropin (ACTH) is a well-known regulator of aldosterone synthesis and secretion as well as angiotensin II and serum potassium levels [6]. ACTH stimulation test (AST) was first established in 1978 [7], when it was shown that PAC responsiveness to ACTH stimulation in PA patients was significantly higher than in essential hypertensive subjects. More recently, studies have assessed the diagnostic accuracy of AST for PA and/or PA subtypes [8,9,10]. However, some studies performed AST during 1-mg dexamethasone suppression (DS) in order to eliminate the action of endogenous ACTH [8,9] while others did not [10]. The effect of 1-mg DS on AST is unclear and assessing this effect is important to perform and precisely evaluate the results of AST. The aim of our study was to elucidate whether PAC responsiveness to ACTH stimulation and the diagnostic accuracy of PA and/or PA subtypes differ between AST with and that without 1-mg DS.

## 2. Results

The clinical characteristics of the 48 patients (Unilateral-PA, 13; Bilateral-PA, 17; Non-PA, 18) in our study are shown in Table 1. The median age (interquartile range) was 49.2 (43.4–56.5) years and 14 patients (29.2%) were male. The PA group showed higher blood pressure compared with the Non-PA group. The Unilateral-PA (U-PA) group had lower potassium levels and higher urinary potassium excretion than the Bilateral-PA (B-PA) and Non-PA groups.

### 2.1. Aldosterone Responsiveness to ACTH Stimulation with and without Dexamethasone Suppression

**Figure 1:**
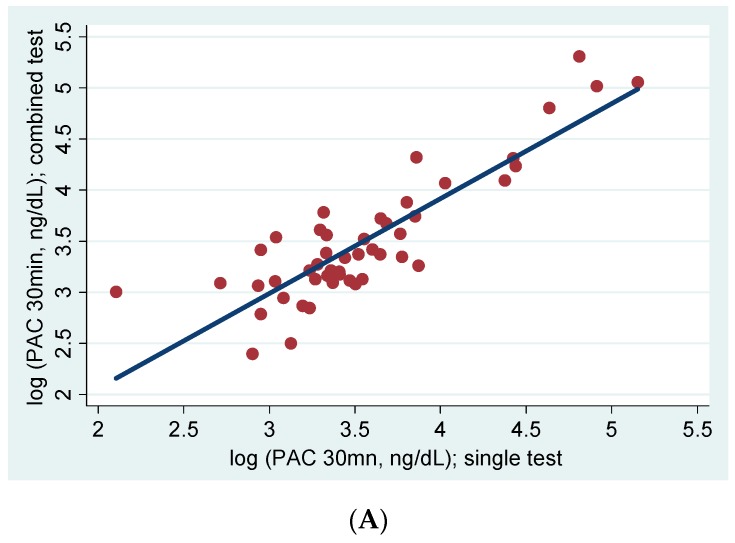

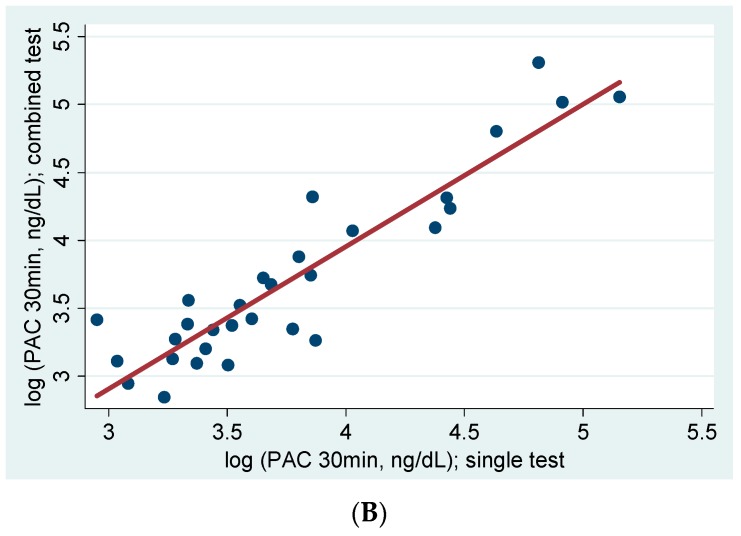
Linear regression analysis of PAC responsiveness to ACTH stimulation between single (AST alone) and combined (AST under 1-mg DS) tests. (**A**) All patients: 30 min, *R*^2^ = 0.75, *p*-value < 0.01; 60 min, *R*^2^ = 0.76, *p*-value < 0.01; (**B**) PA patients: 30 min, *R*^2^ = 0.85, *p*-value < 0.01; 60 min, *R*^2^ = 0.85, *p*-value < 0.01. Abbreviations: PAC, plasma aldosterone concentration; ACTH, adrenocorticotropin; AST, ACTH stimulation test; DS, dexamethasone suppression; PA, primary aldosteronism.

There was no significant difference in PAC responsiveness to ACTH stimulation between single (AST alone) and combined (AST under 1-mg DS) tests in any group (Table 2). The maximum value of PAC following AST in the U-PA group was approximately three times and twice that observed in the Non-PA and B-PA group, respectively. This trend was observed regardless of whether AST was conducted under 1-mg DS or not. Cortisol (F) responsiveness to ACTH stimulation was higher at both 30 and 60 min in the single test than the combined test (Table 2). There were significant differences of the increase ratio of PAC (30/0 min) and the PAC/F ratio (30, 60 min) between these two tests only in the U-PA group (Table A1). A significant positive linear relationship was observed between the log of PAC values for the single test and those for the combined test (Figure 1A). We found similar results when we restricted the patients to those diagnosed with PA (Figure 1B).

### 2.2. Diagnostic Accuracy for PA and the Laterality of Hyperaldosteronism

The single and combined tests showed the similar highest diagnostic accuracy for PA following 30 min of ACTH stimulation (Table 3). Both single and combined tests could also differentiate between the group with lateralization ratio (LR) > 2.6 (LR defined as aldosterone to cortisol ratio from one adrenal gland divided by that from the other adrenal gland) and the group with LR ≤ 2.6 (Table 3). However, neither test could estimate the laterality of hyperaldosteronism as defined by the absolute value of the effluent aldosterone levels from super-selective ACTH loading AVS (SS-ACTH-AVS) (Table 3).

## 3. Discussion

We found that 1-mg DS did not affect the PAC responsiveness to ACTH stimulation. To the best of our knowledge, this is the first study to assess the effect of 1-mg DS on AST results within the same patient. These results raise two important considerations.

Firstly, it is feasible to disregard the effect of 1-mg DS and endogenous ACTH when assessing the PAC responsiveness to exogenous ACTH (25 IU) stimulation. ACTH stimulates aldosterone secretion acutely and transiently through activation in the circulatory system where 11β-hydroxysteroid dehydrogenase type 2 (11βHSD2) is highly expressed [11]. A previous study showed that aldosterone secretion was hypersensitive to even very low dose ACTH (0.003 IU) that was not sufficient to stimulate cortisol secretion [12]. In this context, it has been considered that AST might be a useful tool for PA diagnosis, and several studies have been conducted to clarify the diagnostic accuracy of AST for detecting PA and/or PA subtypes, particularly in Asia [8,9,10]. The majority of these studies performed AST under 1-mg DS in order to eliminate endogenous ACTH-mediated aldosterone hypersecretion [8,9]. However, there has been no evidence for the effect of 1-mg DS on AST results. Previous studies showed that saline-infusion and fludrocortisone tests had a higher positive ratio under 1-mg DS than when performed without 1-mg DS [13,14]. In the present study, we found no difference between single and combined tests when using much higher than physiological levels of ACTH according to the previous studies [9]. The effect of 1-mg DS and endogenous ACTH might be too little for the PAC responsiveness to 25 IU of exogenous ACTH. Meanwhile, we found the significant lower baseline of PAC, and the significant higher PAC/F ratio after the combined test than the single test in the U-PA group. Considering that aldosterone-producing adenoma (APA) had a higher responsiveness to ACTH stimulation than bilateral adrenal hyperplasia [15], we assumed that endogenous ACTH suppression during 1-mg DS might decrease the baseline of PAC and increase the PAC/F ratio in the U-PA group.

Secondly, AST under 1-mg DS may be an effective way to assess not only hyperaldosteronism but also hypercortisolism. Recent studies have suggested that approximately 10–20% cases of APA are complicated with subclinical Cushing’s syndrome [16,17]. Dexamethasone suppression test (DST) is the gold standard to evaluate the cortisol excess [18] and we routinely perform 1-mg DST when there is an adrenal tumor. The lack of significant difference between the results of the two tests in our study suggests that it may be possible to precisely assess PAC responsiveness to ACTH (30, 60 min) and cortisol suppression under 1-mg DS (0 min) at the same time in the combined test. This would reduce the number of required hospital visits to examine AST and 1-mg DST results, reducing the burden on both patients and healthcare providers.

A similar diagnostic accuracy of PA was also observed for both, the single and combined tests. Moreover, based on AST results, it was possible to determine the laterality of hyperaldosteronism defined by LR according to the Japan Endocrine Society (JES) guidelines [4], consistent with previous reports from Asia [8,9]. However, the accuracy of the results was diminished on both tests when aldosterone hypersecretion was defined using the absolute value of the effluent aldosterone levels from adrenal segmental venous in SS-ACTH-AVS. The underestimation of APmicroA (APA which is too small to detect using CT) might cause this discrepancy through misclassification of APA and IHA [19]. A case study of a 48-year-old man conducted by us illustrates this idea (Table A2). We performed a partial adrenalectomy because the absolute value of PAC from the right adrenal inferior branch was >1400 ng/dL although LR was 1.2. The resected adenoma was pathologically confirmed as APA. The patient’s hypertension and endocrine function were completely normalized following the partial adrenalectomy. SS-ACTH-AVS is still not a worldwide examination and there is no consensus about the cut-off value of the effluent aldosterone levels for hyperaldosteronism. However, the discrepancy in our results and this example case imply the importance of considering the absolute value of the effluent aldosterone levels in SS-ACTH-AVS as well as LR in AVS to assess the laterality of hyperaldosteronism. The establishment of the precise evaluation for the laterality in AVS would reveal whether AST is a useful tool for the assessment of the laterality without AVS or not.

The present study has several limitations. First, we did not consider differences in age, sex, and genetic mutations which could affect the renin–angiotensin–aldosterone system [20] and PAC responsiveness to ACTH stimulation [21]. Our results did not depend on such differences because we compared the results of both tests on the same patients, but a stratified study would provide additional information. Second, we “compared” the diagnostic accuracy for PA between single and combined tests in patients with PRA < 1.2 ng/mL/h. It is necessary to restrict the study to patients that are positive for PA screening in order to elucidate the precise ability to detect PA. Finally, the sample size is small, and we could not eliminate a selection bias due to the nature of a retrospective study at a single hospital. Further prospective studies with larger sample sizes from multiple institutions would mitigate these limitations and confirm our findings.

## 4. Materials and Methods

### 4.1. Data Sources and Study Population

We performed a retrospective cohort study using the clinical records at Yokohama Rosai Hospital from 2011 to 2016. We included patients aged 20 years and older with data of AST both with and without 1-mg DS. We excluded cases with adrenal insufficiency, overt Cushing syndrome, and secondary hyperaldosteronism. The Yokohama Rosai Hospital research ethics committee approved this study (Identification code: 24-10, Date of approval: 2 July 2012).

### 4.2. Measurements

Demographic characteristics and history of hypertension were self-reported. We measured weight and height to calculate body mass index (BMI). Antihypertensive drugs were adjusted for several weeks before blood sampling [4]. Morning blood samples were collected after the patient had rested supine for 30 min. Plasma aldosterone concentration (PAC, ng/dL × 27.7 for pmol/L), serum cortisol levels (F, µg/dL × 27.6 for nmol/L), and adrenocorticotropic hormone (ACTH, pg/mL × 0.220 for pmol/L) were measured using radioimmunoassay (RIA), chemiluminescent enzyme immunoassay, and electrochemiluminescent immunoassay, respectively [19,21,22,23]. Plasma renin activities (PRA, ng/mL/h) were measured using RIA [19] until December, 2015, and an enzyme immunoassay since January 2016. Levels of PRA measured using two different assays were confirmed to have a linear relationship (1:1) and hence were treated similarly in subsequent analysis. We employed the plasma aldosterone screening criterion as ARR > 20 for patients [4]. We conducted confirmatory tests to detect PA in patients positive for the screening criterion according to the JES guidelines [4]. “Non-PA” patients were defined as being negative for the screening criterion or any confirmatory tests for PA. Undetectable PRA levels (<0.1 ng/mL/h) were described as “0.05 ng/mL/h” in the statistical analyses. AST was performed as following: 25 IU (0.25 mg) ACTH was injected intravenously at 8:00 a.m. and blood samples were drawn at 0, 30, 60 min after injection to measure serum aldosterone and cortisol levels. When performing AST under 1-mg DS, dexamethasone (1-mg) was administered at 11:00 p.m. the day before AST (single test: AST alone, and combined test: AST under 1-mg DS).

### 4.3. Adrenal Imaging Studies

Thin-section computed tomography (CT) scans of the adrenal glands were performed following intravenous injection of contrast medium. Next, we conducted super-selective ACTH loading AVS (SS-ACTH-AVS) to assess the laterality of hyperaldosteronism for patients diagnosed as PA [4,5,21]. We considered adrenal vein cannulation successful if the effluent cortisol levels before and 30 min after ACTH stimulation were ≥40 µg/dL and ≥200 µg/dL [4,21]. We estimated the laterality of hyperaldosteronism using LR after ACTH stimulation (cut-off value = 2.6) [4].

### 4.4. Statistical Analyses

Fisher's exact probability test for categorical variables and Mann-Whitney’s U test for continuous variables (presented as median (interquartile range)) were used to assess the difference of clinical characteristics among the subgroups (U-PA, B-PA, and Non-PA). The difference of PAC responsiveness to ACTH stimulation between single (AST alone) and combined (AST under 1-mg DS) tests conducted on the same patient was calculated based on Wilcoxon signed-rank test. After we took a log of the PAC value, we performed a linear regression analysis to assess the relationship of PAC responsiveness between these two tests. As an additional analysis, we compared the diagnostic accuracy for PA and the laterality of hyperaldosteronism as assessed by single versus combined test using receiver-operating characteristic (ROC) analysis. We also estimated the diagnostic accuracy for the laterality of hyperaldosteronism defined by the absolute value of the effluent aldosterone concentrations after ACTH stimulation (cut-off value = 1400 ng/dL) [21] instead of LR. All statistical analyses were conducted using Stata software (version 12.1; StataCorp. 2011, College Station, TX, USA). *p*-Value of <0.05 was considered statistically significant.

## 5. Conclusions

We found no difference in PAC responsiveness to ACTH stimulation between AST with and without 1-mg DS. It may be possible to perform AST under both conditions with similar accuracy to detect PA.

## Figures and Tables

**Table 1:** Clinical characteristics ^a^.

Entry	PA (*n* = 30)		Non-PA (*n* = 18)
	U-PA (*n* = 13)	B-PA (*n* = 17)	
Sex (Male), *n* (%)	4 (30.8)	5 (29.4)	5 (27.8)
*p*-Value ^b^		0.62	0.58
Age (years old)	52.0 (39.3–60.4)	48.9 (46.6–54.4)	48.4 (41.9–53.9)
*p*-Value		0.87	0.52
Body mass index (kg/m^2^)	22.3 (20.9–25.3)	25.5 (20.6–28.3)	24.2 (21.3–28.8)
*p*-Value		0.25	0.32
Systolic blood pressure (mmHg)	168.0 (150.0–200.0)	180.0 (167.0–196.0)	141.0 (130.0–190.0)
*p*-Value		0.38	0.05
Diastolic blood pressure (mmHg)	100.0 (90.0–115.0)	110.0 (95.0–120.0)	88.0 (82.0–100.0)
*p*-Value		0.60	0.01
Serum potassium (mmol/L)	3.3 (3.1–3.9)	4.0 (3.7–4.1)	4.0 (3.9–4.2)
*p*-Value		<0.01	<0.01
Serum sodium (mEq/L)	142.0 (141.0–144.0)	141.0 (140.0–142.0)	140.0 (139.0–141.0)
*p*-Value		0.02	<0.01
eGFR (mL/min/1.73/m^2^)	107.8 (97.0–114.0)	107.1 (101.1–111.6)	105.4 (98.1–113.2)
*p*-Value		0.85	0.97
FEk (%)	8.6 (6.2–10.8)	5.0 (3.7–6.2)	4.3 (3.8–5.3)
*p*-Value		0.04	0.01
FEna (%)	0.3 (0.2–0.5)	0.4 (0.3–0.6)	0.4 (0.3–0.6)
*p*-Value		0.48	0.50

Abbreviation: PA, primary aldosteronism; U-PA, unilateral PA; B-PA, bilateral PA; eGFR, estimated glomerular filtration rate; FEk, fractional excretion of potassium, FEna, fractional excretion of sodium. ^a^ Data are presented as median (interquartile range) unless otherwise indicated; ^b^ *p*-Value was calculated for each characteristic in the B-PA and Non-PA group compared to the U-PA group.

**Table 2:** Comparison of PAC and F responsiveness to ACTH stimulation between combined and single tests ^a,b^.

Entry	PA (*n* = 30)				Non-PA (*n* = 18)	
	U-PA (*n* = 13)		B-PA (*n* = 17)			
	Combined	Single	Combined	Single	Combined	Single
PAC (ng/dL), 0 min	19.5	27.9	11.8	12.3	8.3	10.5
	(13.0–24.9)	(15.3–55.9)	(9.8–15.8)	(9.4–16.9)	(7.8–10.1)	(6.9–12.5)
*p*-Value ^c^		0.05		0.62		0.40
PAC (ng/dL), 30 min	69.1	79.5	29.2	33.2	23.2	26.2
	(29.5–122.0)	(38.5–103.0)	(22.4–35.1)	(26.6–39.8)	(20.2–29.1)	(19.1–30.3)
*p*-Value		0.86		0.13		0.62
PAC (ng/dL), 60 min	86.9	77.5	30.7	35.7	27.9	26.6
	(29.7–146.0)	(41.2–121.0)	(22.1–33.9)	(25.3–39.7)	(21.1–31.4)	(20.4–34.9)
*p*-Value		0.55		0.12		0.51
F (µg/dL), 0 min ^d^	1.0	10.4	1.3	6.0	1.0	8.2
	(0.8–1.6)	(8.4–13.0)	(0.8–1.6)	(4.1–7.2)	(0.8–1.8)	(5.7–11)
*p*-Value		<0.01		<0.01		<0.01
F (µg/dL), 30 min	15.2	19.5	17.4	19.4	18.1	20.9
	(13.4–16.9)	(18.3–21.4)	(16.2–21.1)	(17.6–21.5)	(15.2–20.6)	(17.7–24.8)
*p*-Value		<0.01		<0.01		<0.01
F (µg/dL), 60 min	18.3	23.1	21.5	21.8	22.6	24.4
	(17.5–20.9)	(21.6–24.3)	(19.2–26.5)	(21.1–25.5)	(19.6–24.5)	(20.1–27.6)
*p*-Value		<0.01		0.44		0.01

Abbreviations: PAC, plasma aldosterone concentration; F, serum cortisol; U-PA, unilateral PA; B-PA, bilateral PA. Conversion to SI units: PAC, ng/dL × 27.7 for pmol/L; F, µg/dL × 27.6 for nmol/L. ^a^ Data are presented as median (interquartile range) unless otherwise indicated; ^b^ ACTH (25 IU) was iv injected at 8:00 a.m., and blood samples were drawn at 0, 30, 60 min after injection to measure serum aldosterone and cortisol levels. When we perform AST under 1-mg DS, dexamethasone (1-mg) was administered at 11:00 p.m. the day before AST (single test: AST alone, and combined test: AST under 1-mg DS); ^c^ *p*-value was calculated for each value between combined and single test in each group; ^d^ Four and nine patients had serum cortisol levels during the 1-mg DS ≥ 3.0 μg/dL and 1.8 μg/dL (<3.0 μg/dL), respectively.

**Table 3:** ROC analysis of combined and single tests for detecting PA and PA subtypes in patients with PRA < 1.2 ng/mL/h ^a^.

**(A) PA vs. Non-PA**		**Cut-off (ng/dL)**	**Sensitivity**	**Specificity**	**AUC**
Combined	PAC, 30 min	26.1	76.7%	73.3%	0.77
Single	PAC, 30 min	29.1	73.3%	66.7%	0.78
**(B) U-PA vs. B-PA (LR)**		**Cut-off (ng/dL)**	**Sensitivity**	**Specificity**	**AUC**
Combined	PAC, 30 min	41.4	69.2%	82.4%	0.79
Single	PAC, 30 min	38.5	76.9%	70.6%	0.81
**(C) U-PA vs. B-PA (AV)**		**Cut-off (ng/dL)**	**Sensitivity**	**Specificity**	**AUC**
Combined	PAC, 30 min	41.4	47.4%	72.7%	0.54
Single	PAC, 30 min	38.5	52.6%	54.6%	0.54

Abbreviation: AUC, area under the curve; U-PA, unilateral PA; B-PA, bilateral PA; LR, lateralization ratio; AV, absolute value. Conversion to SI units: PAC, ng/dL × 27.7 for pmol/L. ^a^ Diagnostic accuracy for (**A**) PA in patients with PRA < 1.2 ng/mL/h, (**B**) U-PA defined by LR (≥2.6) in patients with PA, and (**C**) U-PA defined by AV (≥1400 ng/mL) in patients with PA. (single test: AST alone, and combined test: AST under 1-mg DS).

## Appendix A

**Table A1:** Comparison of the increase ratio of PAC and the PAC/F ratio after ACTH stimulation between combined and single tests ^a,b^.

Entry	PA (*n* = 30)				Non-PA (*n* = 18)	
	U-PA (*n* = 13)		B-PA (*n* = 17)			
	Combined	Single	Combined	Single	Combined	Single
PAC 30 min/PAC 0 min	3.3	1.8	2.5	2.3	2.7	2.5
	(2.4–4.4)	(1.5–3.0)	(1.9–2.9)	(2.1–2.7)	(2.4–3.0)	(2.1–3.4)
*p*-Value ^c^		0.05		0.49		0.78
PAC 60 min/PAC 0 min	2.9	1.9	2.3	2.3	2.9	2.8
	(2.5–5.0)	(1.7–3.0)	(2.1–2.9)	(2.0–2.9)	(2.7–3.6)	(2.5–3.5)
*p*-Value		0.09		0.52		0.71
PAC/F, 0 min	14.8	3.5	11.8	2.2	7.7	1.3
	(11.5–26.0)	(1.3–5.7)	(6.1–15.7)	(1.5–2.9)	(4.2–11.9)	(1.0–1.5)
*p*-Value		<0.01		<0.01		<0.01
PAC/F, 30 min	4.5	3.8	1.7	1.6	1.3	1.4
	(1.7–8.5)	(1.5–5.3)	(1.4–1.9)	(1.4–2.0)	(1.0–1.8)	(0.9–1.7)
*p*-Value		<0.01		0.94		0.68
PAC/F, 60 min	4.1	3.3	1.4	1.5	1.3	1.1
	(1.5–6.8)	(1.6–4.9)	(1.0–1.6)	(1.1–1.9)	(0.9–1.5)	(0.7–1.6)
*p*-Value		0.05		0.23		0.85

Abbreviations: PAC, plasma aldosterone concentration; F, serum cortisol; U-PA, unilateral PA; B-PA, bilateral PA. Conversion to SI units: PAC, ng/dL × 27.7 for pmol/L; F, µg/dL × 27.6 for nmol/L. ^a^ Data are presented as median (interquartile range) unless otherwise indicated; ^b^ ACTH (25 IU) was iv injected at 8:00 a.m., and blood samples were drawn at 0, 30, 60 min after injection to measure serum aldosterone and cortisol levels. When we perform AST under 1-mg DS, dexamethasone (1-mg) was administered at 11:00 p.m. the day before AST (single test: AST alone, and combined test: AST under 1-mg DS); ^c^ *p*-Value was calculated for each value between combined and single test in each group.

**Table A2:** Results of adrenal venous sampling of a 48-year-old male patient with APmicroA ^a^.

Entry	Right Adrenal Grand				Left Adrenal Grand			LR	Affected Side
	Central Vein	Superior Branch	Lateral Branch	Inferior Branch	Central Vein	Superior Branch	Lateral Branch		
PAC (ng/dL)	465	532	1580	2180	683	734	NA	1.2	Right
Cortisol (μg/dL)	383	449	488	379	457	486	NA		

Abbreviations: PAC, plasma aldosterone concentration; LR, lateralization ratio; NA, not available. Conversion to SI units: PAC, ng/dL × 27.7 for pmol/L; F, µg/dL × 27.6 for nmol/L. ^a^ After ACTH stimulation, blood samples were collected from the superior, lateral, and inferior branches of the right central adrenal vein and the superior and lateral branches of the left central adrenal vein, in addition to the bilateral central adrenal veins. APmicroA was defined as aldosterone-producing adenoma which was too small to detect using computer tomography.
